# Drug Adherence Among Patients With Hypertension and Diabetes Attending Non‐Communicable Disease Corners at Sub‐District Hospitals of Bangladesh in 2024: A Mixed‐Methods Study

**DOI:** 10.1002/hsr2.73515

**Published:** 2026-11-10

**Authors:** Sabrina Mohona, Md. Ahashan Habib, Ifteker Uddin Sakib, Monalisa, Urmila Rahman, Tahmina Shirin, Quazi Ahmed Zaki, Rashedul Hasan

**Affiliations:** ^1^ Directorate General of Health Services Ministry of Health and Family Welfare Dhaka Bangladesh; ^2^ Department of Public Health and Informatics Jahangirnagar University Dhaka Bangladesh; ^3^ Institute of Epidemiology Disease Control and Research (IEDCR) Dhaka Bangladesh; ^4^ Dr. Sirajul Islam Medical College & Hospital Ltd. Dhaka Bangladesh

**Keywords:** Bangladesh, diabetes mellitus, hypertension, medication adherence, MMAS‐8, NCD corner, primary healthcare

## Abstract

**Background:**

Hypertension and diabetes mellitus are major non‐communicable diseases in Bangladesh. Sustained medication adherence is essential for disease control but is influenced by both patient‐ and health‐system factors. This study assessed medication adherence and factors associated with low adherence among patients with hypertension and/or diabetes attending Non‐Communicable Disease (NCD) Corners at sub‐district hospitals in Bangladesh.

**Methods:**

The study employed an exploratory sequential mixed‐methods design conducted at four Upazila Health Complexes in Gopalganj district, Bangladesh. Quantitative data were collected through face‐to‐face interviews with 301 adults receiving treatment for hypertension and/or diabetes for at least 6 months. Medication adherence was assessed using the eight‐item Morisky Medication Adherence Scale. Factors associated with low adherence were identified using Chi‐square tests and multivariable logistic regression. Qualitative data from eight in‐depth interviews with healthcare providers and two focus group discussions with patients were analysed thematically.

**Results:**

Among 301 participants, 76.4% were female, 57.1% were aged 40–59 years, and 68.8% were housewives. Overall, 57.1% had low adherence, 40.2% had moderate adherence, and 2.7% had high adherence. Low adherence was independently associated with being a housewife, missing doses due to drug unavailability or work pressure, and incomplete or absent medicine dispensing from NCD Corners. The strongest association was observed among patients receiving no drugs from the NCD Corner. Qualitative findings complemented these results by identifying medicine stock‐outs, inadequate counselling, travel costs, and misconceptions about lifelong treatment as key barriers to medication adherence.

**Conclusion:**

Low medication adherence was common among patients attending NCD Corners. Strengthening medicine availability, prescription fulfilment, adherence counselling, and patient‐friendly refill mechanisms may improve medication adherence in primary healthcare NCD services in Bangladesh.

**Abbreviations**
AOR: adjusted odds ratio
CI: confidence interval
FGD: focus group discussion
IDI: in‐depth interview
IEDCR: Institute of Epidemiology, Disease Control and Research
IRB: Institutional Review Board
MMAS‐8: Morisky Medication Adherence Scale
NCD: non‐communicable disease
OADs: oral anti‐diabetic drugs
OR: odds ratio
T1DM: type 1 diabetes mellitus
T2DM: type 2 diabetes mellitus
UHC: Upazila Health Complex

## 1 Introduction

Major non‐communicable diseases (NCDs), such as hypertension and diabetes mellitus among the leading causes of morbidity and premature mortality all over the world, especially in low‐ and middle‐income countries (LMICs). The global burden of diabetes has increased substantially over the past three decades, rising from approximately 200 million people in 1990 to 830 million in 2022, with the most rapid increase occurring in LMICs, where diabetes treatment coverage remains comparatively low [1]. Similarly, hypertension is one of the leading modifiable risk factors for premature mortality worldwide and contributes significantly to coronary heart disease, stroke, heart failure, chronic kidney disease, blindness, and disability across the world [2, 3]. Globally, an estimated 1.28 billion adults aged 30–79 years are living with hypertension; however, only about one‐quarter have adequately controlled blood pressure, while nearly two‐thirds of adults living in LMICs have uncontrolled hypertension [4]. Bangladesh has experienced a similar epidemiological transition, with national surveys documenting a substantial and increasing burden of hypertension and diabetes among adults. According to the Bangladesh Demographic and Health Survey (BDHS) 2017–2018, approximately one‐quarter of Bangladeshi adults had hypertension, while diabetes affected about 14% of adults aged 35 years and older [5]. NCDs cause nearly 73% of all deaths in Bangladesh, with cardiovascular diseases being the leading cause of death [6]. When diabetes and hypertension occur together, it poses a significant clinical and economic burden to patients, families, and healthcare systems. These conditions act synergistically to increase cardiovascular risk, accelerate disease progression, complicate clinical management, and worsen long‐term health outcomes [7, 8].

The World Health Organization defines medication adherence as the extent to which a person's medication‐taking behaviour, dietary practices, and lifestyle modifications correspond with the recommendations agreed upon with a healthcare provider [4]. Low medication adherence to prescribed medication is a frequent problem among individuals with hypertension, and it has been shown to worsen blood‐pressure control, increase cardiovascular events, and preventable complications [2]. Similarly, medication adherence among people with diabetes remains highly variable across different populations and continues to be a major barrier to achieving optimal glycaemic control and preventing both microvascular and macrovascular complications [9]. In addition, data from LMICs suggest that low medication adherence is influenced by factors such as treatment cost, medicine access, capacity of the health system, and patient‐level constraints [10]. Polypharmacy, the complex nature and higher cost of treatment, and ongoing long‐term treatment are perceived as increased obstacles to medication adherence for patients with hypertension and diabetes, compared to their peers with hypertension only. Patients living with both hypertension and diabetes often experience additional challenges because they require multiple medications, more complex treatment regimens, lifelong therapy, and more frequent healthcare visits, all of which increase treatment burden and out‐of‐pocket expenditure [7, 8]. Consequently, improving medication adherence requires interventions that address not only individual patient behaviour but also broader health‐system factors, including medicine availability, service organisation, continuity of care, and the quality of primary healthcare services [4, 11].

The establishment of NCD Corners at Upazila Health Complexes in Bangladesh has been introduced to enhance screening, treatment, counselling, referral, and follow‐up for NCDs such as hypertension and diabetes [12]. A policy analysis and a service analysis both highlighted the importance of enhancing the prevention and control of NCDs through the primary health‐care system in Bangladesh [13]. These NCD Corners are the leading primary‐care vehicle across the country to address key concerns regarding the provision of NCD services and access to and equity in NCD management. However, the availability of health services alone is insufficient to ensure effective long‐term disease control. Inconsistent medicine supplies, limited access to prescribed medicines, inadequate counselling, and difficulties in accessing healthcare services may interrupt continuity of treatment and reduce medication adherence [11]. Moreover, even when services are available, adherence may remain suboptimal because of patient‐level barriers, misconceptions regarding lifelong treatment, and difficulties incorporating daily medication use into routine life [14].

Several individual and health‐system factors were found to be important contributors to patient adherence to medicines; however, qualitative studies have revealed that patients' beliefs, financial considerations, family responsibilities, provider communication, and access to medicines interact to contribute to long‐term adherence behaviour [15]. Previous studies on medication adherence in Bangladesh have mostly been conducted in rural or urban populations of diabetes patients, or focused on the relation between adherence and other factors like patient perceptions and quality of life [14, 16]. These studies have offered important insights into the behavioural and psychosocial factors related to adherence, but have not fully examined adherence in the primary‐care NDC Corners programme in the country, where medicine availability, prescription filling, and continuity of care could significantly impact adherence. The studies already conducted in Bangladesh have only studied diabetes, urban specialised clinics, health‐related quality of life, or single designs of quantitative/qualitative studies [14, 16]. Thus, there is limited evidence on how well individuals with hypertension and/or diabetes take their medication during routine health visits in the sub‐district hospitals where the national NCD policy is housed, which we call NCD Corners. Additionally, few studies have tested patient‐, disease‐, and health‐system‐related factors of adherence simultaneously and incorporated quantitative results and patient and health‐system perspectives. Specifically, little information is available about the effect of medicine availability, prescription fulfilment, quality of counselling, and continuous provision of the core services provided in NCD corners on long‐term adherence. All of these gaps in evidence need to be addressed to drive evidence‐based interventions and build needed capacity in Bangladesh's primary care for chronic disease management.

Therefore, this study aimed to assess medication adherence among patients with hypertension and/or diabetes attending NCD Corners at sub‐district hospitals in Bangladesh in 2024. The study also sought to identify patient, disease, and health‐system‐related factors associated with poor medication adherence among adults diagnosed with hypertension and/or diabetes. Furthermore, this mixed‐methods study sought to help contextualise the quantitative findings, identify practical service delivery barriers, and provide evidence to guide the development of supporting service delivery enhancements of medicine availability, adherence counselling, follow‐up systems, and effectiveness of primary care NCD services in Bangladesh.

## 2 Methods

### 2.1 Study Design and Setting

The study employed an exploratory sequential mixed‐methods design with a duration of 6 months from 14 August 2024 to 15 February 2025, at four Upazila Health Complexes in Gopalganj district, Bangladesh. These are the selected facilities that had NCD corners in sub‐district hospitals. The NCD Corners had been established in all Upazila Health Complexes of the district except Gopalganj Sadar Upazila Health Complex. Therefore, the study was conducted in the UHCs of Kotalipara, Kashiani, Tungipara, and Muksudpur. Gopalganj District was purposively selected because it was the working area of the principal investigator, and it was possible to have logistical feasibility, as well as easy access to the study population and coordinating data collection.

The study comprised two sequential phases. In the first (qualitative) phase, in‐depth interviews (IDIs) with healthcare providers and focus group discussions (FGDs) were conducted with patients attending the NCD Corners at Tungipara and Kashiani UHCs to explore perceptions of medication adherence and identify reasons for low medication adherence to prescribed medications. Based on the results of the qualitative phase, four Upazila Health Complexes (UHCs) were selected in the second phase for the qualitative cross‐sectional survey on eligible patients. The quantitative data were collected from 15 September to 25 December 2024 and focused on understanding the prevalence of low medication adherence and identifying the factors contributing to low medication adherence.

The phases were linked sequentially, with the qualitative findings providing contextual understanding of adherence barriers for the subsequent quantitative phase. Integration occurred during interpretation by comparing quantitative findings with qualitative themes to identify convergence and contextualise statistical associations.

### 2.2 Study Population

For the quantitative component, adults aged 18 years or older who had been diagnosed with hypertension and/or type 1 diabetes mellitus (T1DM) and/or type 2 diabetes mellitus (T2DM) by a registered physician and had received treatment for at least 6 months were eligible to participate. The 6‐month treatment criterion was used for medication adherence measurement to ensure long‐term measures were taken to gain sufficient exposure to long‐term use of the medication. Complications related to hypertension and/or diabetes, pregnant women, and critically ill patients were excluded. For the qualitative part of the study, Tungipara and Kashiani Upazila Health Complexes were purposively selected because they were included in the exploratory qualitative phase of the study. Eight in‐depth interviews (IDIs) were conducted with healthcare providers involved in NCD Corner services, including the Upazila Health and Family Planning Officer, Medical Officer responsible for the NCD Corner, nurses involved in NCD patient care, and pharmacists responsible for medicine dispensing. Purposive sampling was used to recruit participants for the focus group discussions to ensure representation of both male and female patients and to capture a range of experiences related to medication adherence and NCD Corner services. Two focus group discussions were conducted: one with the women and the other with the men who attended NCD Corners.

### 2.3 Sample Size and Sampling

The minimum sample size for the quantitative part of the study has been calculated by the single population proportion formula, *n* = (*z* ^2^ *pq*)/*d* ^2^, where: *n* = sample size, *z* = level of significance, *p* = estimated proportion, *q* = the complement of *p*, *d* = margin of error (5%), and *p* = estimated prevalence of the outcome. A prevalence of 26% (*p* = 0.26) [17] was assumed because it provides the maximum sample size and was consistent with the Bangladesh National STEPS Survey 2018 national prevalence estimate. The originally planned sample size was 329 participants. A total of 360 eligible patients were approached, of whom 301 completed the study and were included in the final analysis, yielding a response rate of 83.6% (Figure 1). Eligible patients attending the selected NCD Corners during the study period were identified according to the study eligibility criteria and selected using a simple random sampling technique until the required sample size was achieved. The reasons for non‐participation or exclusion among the 59 patients not included in the final analysis were not specified in the original study records available for this report. In the qualitative part of the study, purposive sampling was used to select healthcare providers and patients who were experienced and knowledgeable about the NCD Corner, as well as to capture different experiences of patients using the corner for medicines and perceived barriers to medicines use.

**FIGURE 1:**
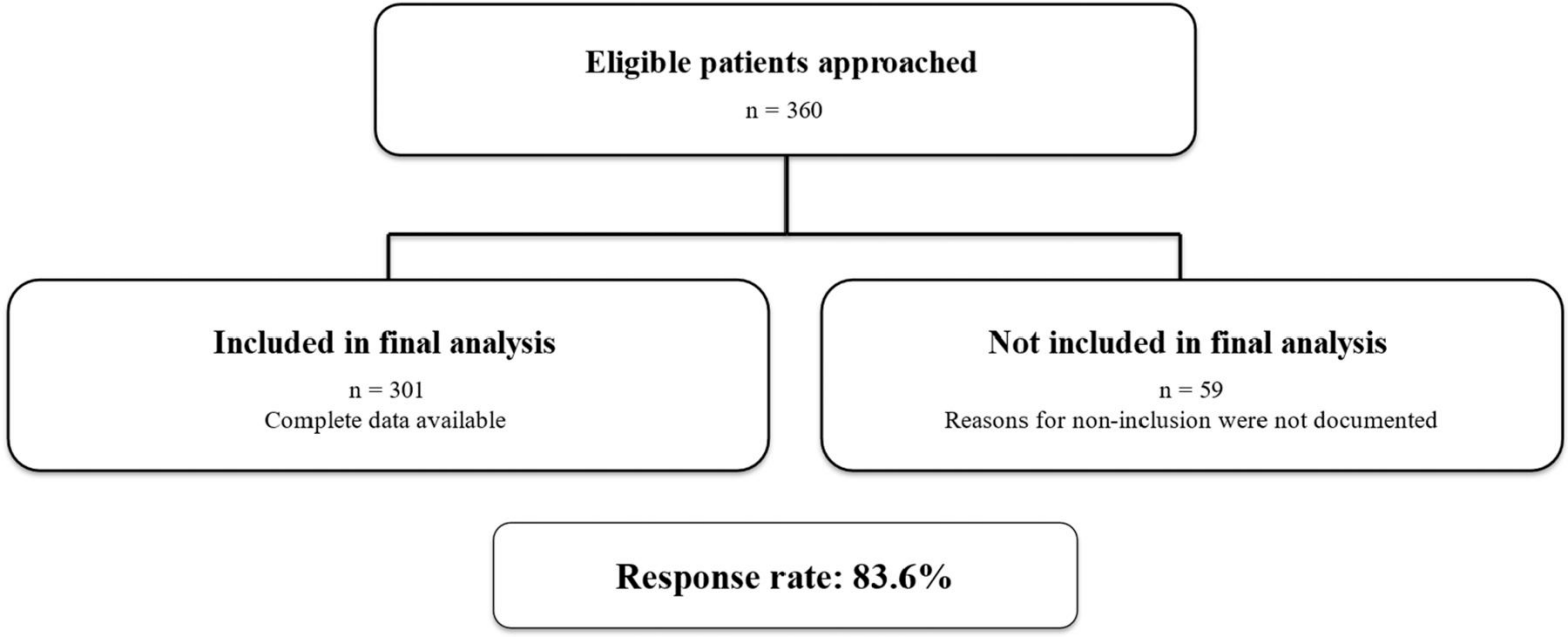
Participant flow diagram.

### 2.4 Data Collection Tools and Procedures

A face‐to‐face, interviewer‐administered, pre‐designed, structured, and pre‐tested Bangla‐translated questionnaire was used to collect quantitative data. The data were collected from 15 September 2024 to 25 December 2024. The translation was carried out based on the forward–backward approach for linguistic accuracy and conceptual equivalence, and was pre‐tested in Chitolmari Upazila Health Complex of Bagerhat District. The findings from the pre‐test were used to refine the questionnaire, improving its clarity, comprehensibility, and cultural appropriateness. The translation and pre‐testing procedures were guided by a previous study conducted in Bangladesh using the MMAS‐8 [18]. The internal consistency of the MMAS‐8 was assessed during the pre‐test, and Cronbach's alpha indicated acceptable internal consistency. The questionnaire collected information on socio‐demographic factors, clinical factors, medication adherence, missed doses, distance travelled from participants' homes, and their medicines prescribed and dispensed at the NCD Corner. Medication dispensing pattern was categorised according to the proportion of prescribed medicines dispensed at the NCD Corner and whether the prescribed doses were supplied in full or incompletely. The categories were defined according to the original questionnaire and coding framework to distinguish participants who received no medicines from the NCD Corner from those who did not obtain their medicines from this facility. Where available, medical records were examined to check against the participants' diagnosis and history of treatment.

Medication adherence was assessed using the eight‐item Morisky Medication Adherence Scale (MMAS‐8) [19]. The MMAS‐8 is a validated self‐reported instrument consisting of eight items designed to assess adherence to prescribed medications among patients with chronic diseases. The first seven items were dichotomously (Yes/No) answered, while the eighth item was on a 5‐point Likert‐type response scale, with the scores applied using the standard MMAS‐8 scoring algorithm. The total scores ranged from 0 to 8, with higher scores indicating more adherence to medication. The score was graded as low adherence (< 6), moderate adherence (6 to < 8), or high adherence (8) using the standard‐defined classifications of the MMAS‐8 [18]. The primary endpoint for quantitative analysis was to measure medication adherence. Moderate and high adherence were grouped and compared to low adherence with bivariate and multivariate logistic regression, as only a few subjects (*n* = 8) had a high adherence level, leading to statistical instability of a comparable analysis of three adherence levels.

A semi‐structured interview guide was developed for the in‐depth interviews (IDIs) and focus group discussions (FGDs) based on a review of the literature and the study objectives. Eight in‐depth interviews were conducted with healthcare providers, and two focus group discussions with NCD patients visiting NCD Corners were evaluated. For the first FGD, 10 women participated in the female FGD, while eight men participated in the male FGD. Nine men were invited to the male FGD, and one participant withdrew during the discussion. Through interviews and conversations with them, the assessments included areas such as health, adherence to medication prescriptions, accessing health care, treatment barriers, and counselling, among other topics. The interviews and focus group discussions took place in private rooms in the participating health facilities in Bangla with trained public health researchers to ensure anonymity and facilitate open conversation. The researchers had no prior relationship with the participants. With participants' written informed consent, all sessions were audio‐recorded. In addition, detailed field notes were taken during each session to capture contextual information, non‐verbal cues, and observations that could not be obtained from the audio recordings but were considered important for qualitative data analysis. The IDIs lasted approximately 10–15 min, while the FGDs lasted approximately 30–45 min. The audio recordings were transcribed in Bangla and translated into English for analysis.

### 2.5 Data Analysis

The quantitative data were analysed using Microsoft Excel 2020 to utilise them for data management, and then statistical data analysis was done on them using STATA version 17. Socio‐demographic and clinical characteristics of the participants and medication adherence scores were summarised using descriptive statistics. The Pearson's chi‐square test was used to examine the associations between the independent variables and medication adherence. Medication adherence was dichotomised as low (MMAS‐8 score < 6) and moderate/high (MMAS‐8 score ≥ 6) for use in regression analyses [18]. Bivariate analyses revealed factors with a *p*‐value of < 0.05, and these were used for multivariable binary logistic regression to identify factors associated with low medication adherence while controlling for potential confounding variables. A significance‐based variable‐selection approach has also been used in published cross‐sectional research on antihypertensive medication adherence [20]. The reporting of the quantitative cross‐sectional component was reviewed against the Strengthening the Reporting of Observational Studies in Epidemiology (STROBE) recommendations [21]. The dispensing category ‘All drugs with complete dose’ was used as the reference category because it represents complete fulfilment of prescribed medication at the NCD Corner. The other categories were retained separately because they represent different patterns of medication dispensing and access. The results are presented as crude and adjusted odds ratios and the corresponding 95% confidence intervals. All statistical tests were two‐tailed, and statistical significance was set at *p* < 0.05.

Thematic analysis was used to analyse the qualitative data and to record it verbatim. Three researchers were involved in coding the qualitative data, and no coding disagreements were reported. Data saturation was not assessed. Multiple readings of the transcripts were used to familiarise oneself with the data before systematic coding. Data were grouped into similar codes to establish general categories and themes related to medication adherence behaviour, whether medicines were available, counsellor, system factors, and patient perspectives. The qualitative and quantitative components were analysed separately and subsequently integrated at the interpretation stage. Findings from the in‐depth interviews and focus group discussions were compared and triangulated with the quantitative results to enhance interpretation and provide a comprehensive understanding of the barriers and facilitators influencing medication adherence. A joint display was developed to present related quantitative findings, qualitative themes, and integrated interpretations side by side. The reporting of the qualitative component, including the in‐depth interviews and focus group discussions, was reviewed against the Consolidated Criteria for Reporting Qualitative Research (COREQ) checklist [22].

### 2.6 Ethical Approval

Institute of Epidemiology, Disease Control and Research (IEDCR) Institutional Review Board (IRB) gave us ethical clearance before data collection (Memo no‐IEDCR/IRB/2021/04). Informed consent forms were signed by all participants after carefully informing them about the aim of the study, the procedures to be undertaken, potential risks, and benefits of participation, with emphasis on volunteerism. Participants were informed that they were free to opt out of the study at any time, and if they did not desire to participate in the study, this would not place any special burden on the healthcare service they received. To ensure participant confidentiality, each participant was assigned a unique identification number; all study data were stored in a password‐protected database accessible only to the research team; and all personal identifiers were removed from the dataset before analysis. All study procedures were conducted in accordance with the ethical principles of the Declaration of Helsinki.

## 3 Results

### 3.1 Socio‐Demographic and Clinical Characteristics of the Study Participants

A total of 360 eligible patients were approached at the NCD Corners across four sub‐district hospitals in the Gopalganj district, and 301 completed the study and were included in the final analysis, resulting in a response rate of 83.6%. Most participants were female (76.4%) and aged 40–59 years (57.1%). Housewives comprised the largest occupational group (68.8%). The most common educational attainment was primary education (34.2%), followed by no formal education (29.9%). Regarding disease status, 37.9% of participants had hypertension, 30.9% had diabetes, and 31.2% had both diabetes and hypertension. More than half of the participants (55.8%) lived more than 5 km away from an NCD Corner (Table 1).

**TABLE 1:** Socio‐demographic and clinical characteristics of patients attending NCD Corners, Gopalganj, Bangladesh, 2024.

Variables	Frequency (*n*)	Percentage (%)
Age (years)		
18–39	25	8.3
40–59	172	57.1
> = 60	104	34.6
Gender		
Female	230	76.4
Male	71	23.6
Education level		
Illiterate	44	14.6
No formal education	90	29.9
Primary	103	34.2
Secondary and above	64	21.3
Occupation		
Housewife	207	68.8
Farming	26	8.6
Business	12	4.0
Service	9	3.0
Day labour	8	2.7
Retired	39	13.0
Monthly household expenditure (BDT)		
< = 15,000	128	42.5
15,001–30,000	158	52.5
> 30,000	15	5.0
Disease status		
Diabetes	93	30.9
Hypertension	114	37.9
Both diabetes and hypertension	94	31.2
Duration of disease		
< = 1 year	55	18.3
> 1–5 years	101	33.6
> 5–10 years	92	30.6
> 10 years	53	17.6
Distance from NCD Corner		
< =5 km	133	44.2
> 5 km	168	55.8

*Note:* Percentages were calculated using total sample size (*N* = 301). Percentages may not total 100 because of rounding.

### 3.2 Medication Adherence Level Among Participants

Based on the MMAS‐8 scoring system, 172 participants (57.1%) had low medication adherence, 121 (40.2%) had moderate adherence, and only eight (2.7%) had high adherence. Thus, fewer than half of the patients demonstrated moderate‐to‐high medication adherence. Analysis of individual MMAS‐8 items showed that 49.5% of participants reported sometimes forgetting to take their medicines for hypertension and/or diabetes, and 36.5% reported missing their medication on at least 1 day during the previous 2 weeks. Although 85.7% reported taking their medicines on the day before the interview, 22.6% reported feeling hassled by taking daily medication and 14.0% reported discontinuing their medication when they perceived their condition to be under control (Figure 2).

**FIGURE 2:**
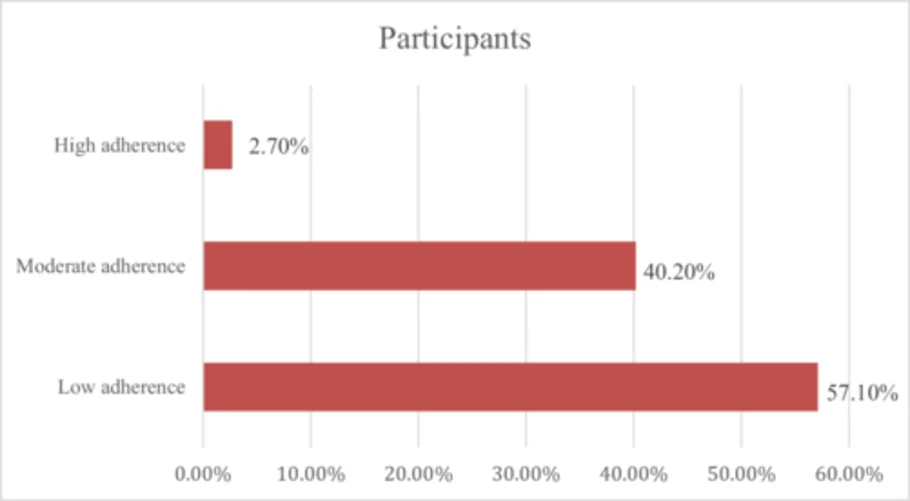
Medication adherence level among patients attending NCD Corners.

### 3.3 Factors Associated With Low Medication Adherence

The bivariate analysis showed that education level, occupation, distance from the NCD Corner, reasons for missed doses, and drug dispensing pattern were significantly associated with low medication adherence. Participants with no formal education had the highest proportion of low adherence (72.2%). Low adherence was also most common among day labourers (75.0%) and housewives (61.4%), whereas retired participants showed the lowest proportion of low adherence (35.9%). Patients living more than 5 km from the NCD Corner had a higher proportion of low adherence compared with those living within 5 km (64.9% vs. 47.4%, *p* = 0.002). Reasons for missing doses were strongly associated with adherence (*p* < 0.001). Low adherence was highest among participants reporting drug unavailability as the reason for missed doses (77.3%), followed by work pressure (61.7%). Drug dispensing pattern was also strongly associated with adherence (*p* < 0.001). Low adherence was lowest among patients who received all prescribed drugs at the complete prescribed doses (27.5%) and highest among those who received no drugs from the NCD Corner (93.8%). Age, gender, monthly expenditure, disease status, and duration of disease were not significantly associated with adherence in the bivariate analysis (Figure 3) (Table 2).

**FIGURE 3:**
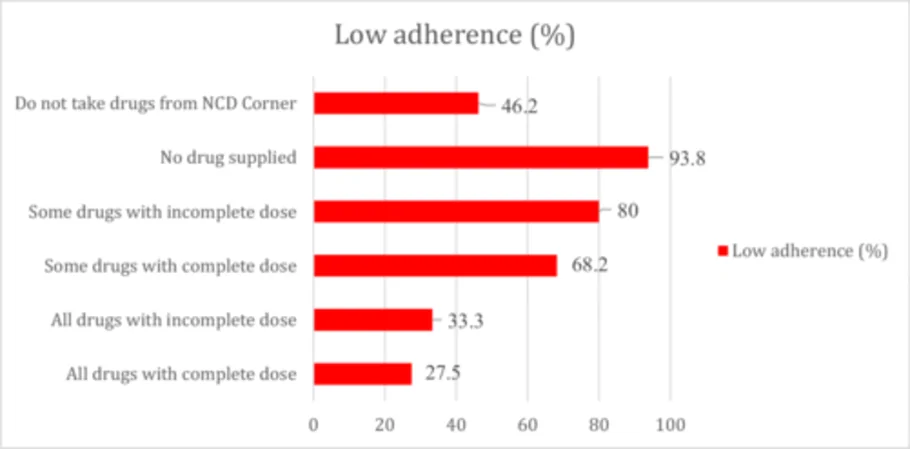
Low medication adherence by drug dispensing pattern.

**TABLE 2:** Factors associated with low medication adherence among patients attending NCD Corners, Gopalganj, Bangladesh, 2024.

Variables	Low adherence *n* (%)	Moderate/high adherence *n* (%)	*p*‐value
Age (years)			
18–39	19 (76.0)	6 (24.0)	0.127
40–59	97 (56.4)	75 (43.6)	
> = 60	56 (53.9)	48 (46.2)	
Gender			
Female	133 (57.8)	97 (42.2)	0.666
Male	39 (54.9)	32 (45.1)	
Education level			
Illiterate	18 (40.9)	26 (59.1)	0.001
No formal education	65 (72.2)	25 (27.8)	
Primary	59 (57.3)	44 (42.7)	
Secondary and above	30 (46.9)	34 (53.1)	
Occupation			
Housewife	127 (61.4)	80 (38.7)	0.026
Farming	16 (61.5)	10 (38.5)	
Business	4 (33.3)	8 (66.7)	
Service	5 (55.6)	4 (44.4)	
Day labour	6 (75.0)	2 (25.0)	
Retired	14 (35.9)	25 (64.1)	
Disease status			
Diabetes	55 (59.1)	38 (40.9)	0.349
Hypertension	69 (60.5)	45 (39.5)	
Both	48 (51.1)	46 (48.9)	
Distance from NCD Corner			
< =5 km	63 (47.4)	70 (52.6)	0.002
> 5 km	109 (64.9)	59 (35.1)	
Reason for missed dose			
Forgot	29 (41.4)	41 (58.6)	< 0.001
At wish	17 (40.5)	25 (59.5)	
Drug unavailable	68 (77.3)	20 (22.7)	
Work pressure	58 (61.7)	36 (38.3)	
Drug dispensing pattern			
All drugs with complete dose	11 (27.5)	29 (72.5)	< 0.001
All drugs with incomplete dose	16 (33.3)	32 (66.7)	
Some drugs with complete dose	58 (68.2)	27 (31.8)	
Some drugs with incomplete dose	48 (80.0)	12 (20.0)	
No drug supplied	15 (93.8)	1 (6.3)	
Do not take drugs from NCD Corner	24 (46.2)	28 (53.9)	

*Note: p*‐values were obtained from Chi‐square tests. Moderate and high adherence were combined for comparison with low adherence.

### 3.4 Multivariable Logistic Regression Analysis of Factors Associated With Low Medication Adherence

After adjustment for variables that were statistically significant in the bivariate analysis, occupation, reason for missed dose, and medication dispensing pattern remained significantly associated with low medication adherence. Housewives had significantly higher odds of low adherence than retired participants (AOR 2.62, 95% CI 1.15–5.98, *p* = 0.022). Participants who missed doses because of drug unavailability had substantially higher odds of low adherence compared with those who reported forgetting to take their medicines (AOR 5.87, 95% CI 2.62–13.16, *p* < 0.001). Similarly, missing doses due to work pressure were also independently associated with low adherence (AOR 2.26, 95% CI 1.08–4.70, *p* = 0.030).

Medication dispensing pattern showed the strongest adjusted association with low adherence. Compared with patients who received all prescribed drugs in complete doses, those who received only some prescribed drugs in complete doses had significantly higher odds of low medication adherence (AOR 4.92, 95% CI 1.95–12.42, *p* = 0.001). The likelihood of low medication adherence increased further among patients who received only some prescribed medications in incomplete doses (AOR 10.04, 95% CI 3.33–30.29, *p* < 0.001), and was highest among patients who received no medications from the NCD Corner (AOR 29.57, 95% CI 3.21–272.72, *p* = 0.003). In contrast, education level and distance from the NCD Corner were no longer statistically significant in the adjusted model (Figure 4) (Table 3).

**FIGURE 4:**
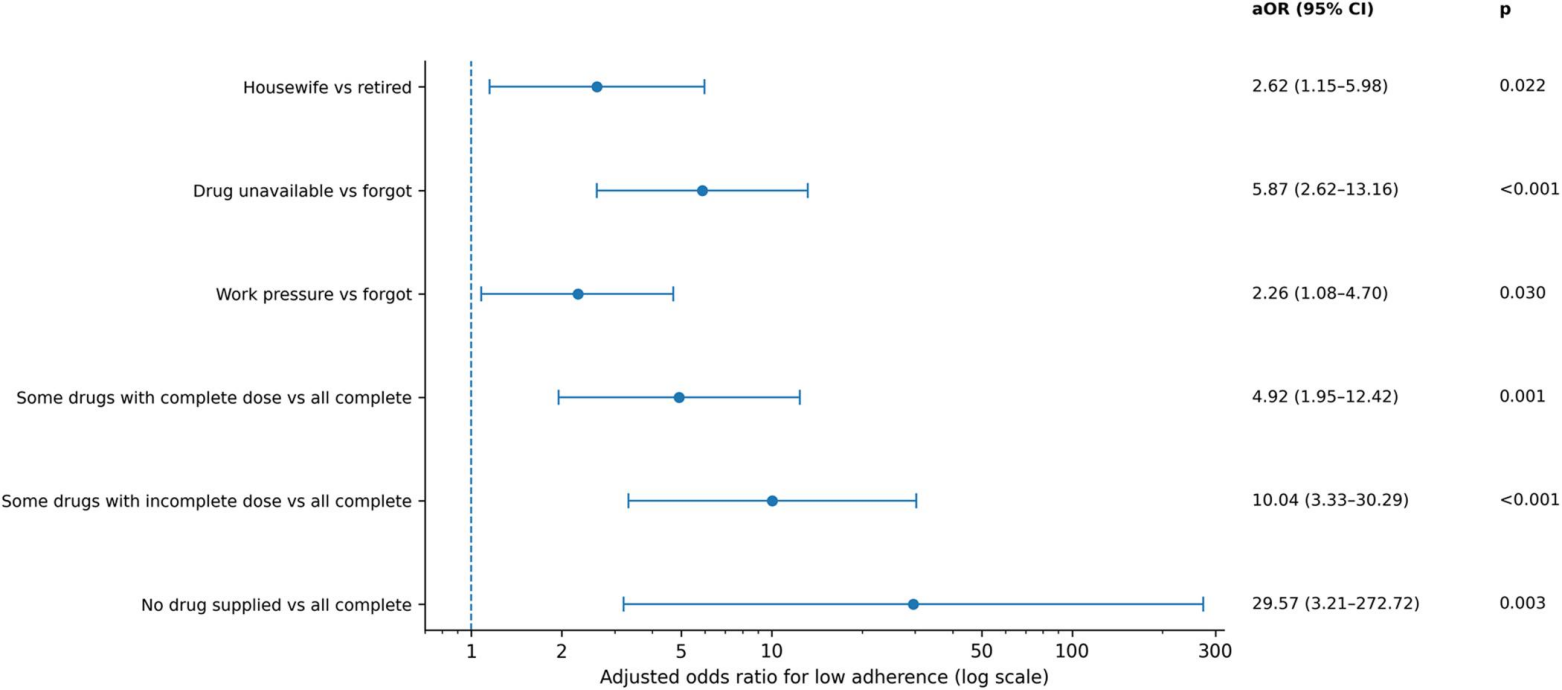
Adjusted predictors of low medication adherence.

**TABLE 3:** Multivariable logistic regression analysis of factors associated with low medication adherence.

Variables	Low adherence *n* (%)	Adjusted odds ratio	95% CI	*p*‐value
Education level				
Illiterate	18 (40.9)	0.76	0.29–2.00	0.578
No formal education	65 (72.2)	1.32	0.55–3.14	0.530
Primary	59 (57.3)	1.30	0.61–2.76	0.496
Secondary and above	30 (46.9)	Reference	—	—
Occupation				
Housewife	127 (61.4)	2.91	0.84–10.10	0.092
Farming	16 (61.5)	3.45	0.29–41.27	0.328
Business	4 (33.3)	1.21	0.22–6.81	0.826
Service	5 (55.6)	1.81	0.32–10.09	0.501
Day labour	6 (75.0)	2.62	1.15–5.98	0.022
Retired	14 (35.9)	Reference	—	—
Distance from NCD Corner				
< = 5 km	63 (47.4)	Reference	—	—
> 5 km	109 (64.9)	1.43	0.80–2.54	0.228
Reason for missed dose				
Forgot	29 (41.4)	Reference	—	—
At wish	17 (40.5)	1.24	0.51–3.03	0.641
Drug unavailable	68 (77.3)	5.87	2.62–13.16	< 0.001
Work pressure	58 (61.7)	2.26	1.08–4.70	0.030
Drug dispensing pattern				
All drugs with complete dose	11 (27.5)	Reference	—	—
All drugs with incomplete dose	16 (33.3)	1.06	0.38–2.91	0.916
Some drugs with complete dose	58 (68.2)	4.92	1.95–12.42	0.001
Some drugs with incomplete dose	48 (80.0)	10.04	3.33–30.29	< 0.001
No drug supplied	15 (93.8)	29.57	3.21–272.72	0.003
Do not take drugs from NCD Corner	24 (46.2)	2.48	0.89–6.93	0.081

*Note:* Outcome variable was low medication adherence, defined as MMAS‐8 score < 6. CI, confidence interval.

### 3.5 Integration of Quantitative and Qualitative Findings

The quantitative and qualitative findings were integrated during interpretation to examine convergence and complementarity and to contextualise selected quantitative results. As shown in Table 4, both components highlighted medicine availability and prescription fulfilment as relevant concerns in medication adherence. In the quantitative analysis, receiving no medication from the NCD Corner was associated with higher odds of low medication adherence than receiving all prescribed medicines in complete doses (AOR 29.57, 95% CI 3.21–272.72; *p* = 0.003). Participants receiving only some prescribed medicines, whether in complete or incomplete doses, also had higher odds of low adherence. Qualitative accounts describing difficulties associated with medicine availability and the financial and time burdens of travelling to health facilities without obtaining required medicines provided contextual insight into these findings. Nevertheless, the wide confidence interval for receiving no medication indicates substantial uncertainty about the magnitude of the association.

**TABLE 4:** Joint display integrating quantitative and qualitative findings on medication adherence among patients attending NCD Corners in Gopalganj, Bangladesh, 2024.

Quantitative finding	Qualitative finding	Integrated interpretation
Participants receiving no medication from the NCD Corner had higher odds of low medication adherence than those receiving all prescribed medicines in complete doses (AOR 29.57; 95% CI 3.21–272.72; *p* = 0.003). Participants receiving some prescribed medicines in complete doses (AOR 4.92; 95% CI 1.95–12.42; *p* = 0.001) or some medicines in incomplete doses (AOR 10.04; 95% CI 3.33–30.29; *p* < 0.001) also had higher odds of low adherence.	Healthcare providers described difficulties supporting patients when prescribed medicines were unavailable. Patients described the financial and time burden of travelling to a facility without receiving the medicines they needed.	The quantitative and qualitative findings jointly highlight medicine availability and prescription fulfilment as important service‐delivery concerns. The wide confidence interval for receiving no medication indicates substantial uncertainty about the magnitude of this association, warranting cautious interpretation.
Drug unavailability (AOR 5.87; 95% CI 2.62–13.16; *p* < 0.001) and work pressure (AOR 2.26; 95% CI 1.08–4.70; *p* = 0.030) were associated with low adherence compared with forgetting medication.	The qualitative findings described medicine‐access barriers and the financial and time burdens reported by patients.	The quantitative findings identify reported reasons for missed doses associated with low adherence. The qualitative findings provide complementary context for medicine‐access barriers. A specific qualitative explanation of work pressure should be included only if supported by the interview or FGD data.
Low adherence was more common among participants living more than 5 km from an NCD Corner than among those living within 5 km (64.9% vs. 47.4%; *p* = 0.002). The association was not statistically significant after adjustment (AOR 1.43; 95% CI 0.80–2.54; *p* = 0.228).	Patients described the financial and time burden of travelling to a facility, particularly when they did not receive the medicines they needed.	The qualitative accounts contextualise the bivariate association between distance and adherence. However, the adjusted analysis did not establish a statistically significant independent association between distance and low adherence. The findings do not establish a causal pathway between distance and adherence.

The quantitative analysis also identified drug unavailability and work pressure as reported reasons for missed doses associated with low adherence. Qualitative accounts of medicine‐access difficulties complemented the finding concerning drug unavailability by illustrating the practical challenges patients may encounter. However, the qualitative findings should not be interpreted as explaining the association with work pressure unless this theme is directly supported by the interview or focus‐group data. Distance from the NCD Corner was associated with low adherence in the bivariate analysis, but the association was not statistically significant after adjustment (AOR 1.43, 95% CI 0.80–2.54; *p* = 0.228). Participants' accounts of travel‐related financial and time burdens provided relevant context, but the adjusted analysis did not establish an independent statistical association between distance and low adherence. Overall, the joint display demonstrates how the qualitative findings contextualised selected quantitative results while highlighting the uncertainty and interpretive limits of the available evidence.

## 4 Discussion

This mixed‐methods study found that low medication adherence was widespread among patients with hypertension and/or diabetes attending NCD Corners at sub‐district hospitals in Gopalganj, Bangladesh. Among the 301 participants, 57.1% had low medication adherence, 40.2% had moderate adherence, while high medication adherence was uncommon according to the MMAS‐8, at around 2.7%. This finding is consistent with previous systematic‐review evidence showing that adherence to antihypertensive therapy remains suboptimal in many settings [2]. Similarly, suboptimal adherence to antidiabetic medication has been consistently reported across diverse healthcare settings [9]. People with diabetes also indicate that medication adherence is closely linked with patient outcomes and health‐related quality of life [16]. Comparable evidence from the Eastern Democratic Republic of Congo found that 56.4% of 211 patients receiving treatment for hypertension had medication non‐adherence, with adherence associated with selected health‐related quality‐of‐life domains [23]. However, differences in disease populations and healthcare settings should be considered when comparing these findings with the present study. Recent evidence from Bangladesh further demonstrates that suboptimal medication adherence remains a persistent challenge across different levels of healthcare. A study conducted among patients attending rural sub‐district NCD services reported that 40.4% achieved high medication adherence, whereas 33.2% had low adherence despite receiving routine chronic disease care, indicating that adherence problems persist even within established NCD programmes [24]. Similarly, a tertiary hospital‐based study among hypertensive patients reported good adherence in 54.8% of participants and identified forgetfulness and busy work schedules as the most common reasons for missed medication [25]. Although direct comparison should be interpreted cautiously because of differences in study populations, disease profiles, and adherence measurement tools, these findings consistently indicate that medication adherence remains suboptimal across diverse healthcare settings in Bangladesh. Unlike previous Bangladeshi studies that primarily investigated hypertension or diabetes separately, the present mixed‐methods study evaluated patients with hypertension, diabetes, and coexisting disease attending rural NCD Corners, providing complementary evidence on both behavioural and health‐system determinants of medication adherence.

The present findings suggest that low adherence cannot be explained only by individual forgetfulness or patient choice. A systematic review on hypertension treatment in developing countries identified treatment cost, limited counselling, access barriers, and patient beliefs as important determinants of adherence [3]. Similarly, a rural Bangladesh study reported financial problems (51.7%), forgetfulness (36.6%), and drug unavailability (23.7%) as perceived reasons for low‐to‐moderate adherence [24]. Consistent with this broader evidence, a recent systematic review of 26 studies involving more than 6500 participants across 10 sub‐Saharan African countries identified socioeconomic barriers, treatment‐related factors, health‐system limitations, and sociocultural influences as determinants of diabetes medication adherence [26]. Although this review focused on diabetes in a different regional context, it reinforces the importance of considering medication adherence as a multidimensional issue shaped by both patient‐related and health‐system factors. In this study, nearly half of participants reported sometimes forgetting medicines, and a substantial proportion reported missed doses within the previous 2 weeks. These behavioural barriers occurred alongside service‐level constraints, particularly irregular medicine availability and incomplete prescription fulfilment. These findings highlight the relevance of behavioural and health‐system barriers, although the association between reported missed‐dose reasons and MMAS‐8 adherence in our study warrants cautious interpretation because of potential conceptual overlap. The strongest quantitative finding was the association between medication access at NCD Corners and low adherence. Participants who missed doses because medicines were unavailable had markedly higher odds of low adherence. Because of the cross‐sectional design, these associations do not establish whether medicine unavailability preceded low medication adherence. The findings should therefore be interpreted as exploratory associations rather than evidence of causal effects. Patients who received only some medicines or no medicines from the NCD Corner also had substantially higher adjusted odds of low adherence than those receiving all prescribed medicines in complete doses. This supports evidence that access to essential medicines is a core requirement for continuous NCD care at primary‐care level [11]. Although receiving no medication from the NCD Corner was strongly associated with low medication adherence (AOR 29.57, 95% CI 3.21–272.72), the wide confidence interval indicates substantial uncertainty regarding the magnitude of this association. This imprecision may partly reflect the small number of participants in this dispensing category and the limited distribution of adherence outcomes. Therefore, the point estimate should be interpreted cautiously and should not be considered a precise measure of the strength of the association, as sparse data can affect the reliability of logistic regression estimates [27]. These findings indicate an association rather than a causal effect.

The qualitative findings reinforced this interpretation. Providers reported that they could not adequately support patients when prescribed medicines were unavailable, while patients described the financial and time burden of travelling to a facility and then failing to receive the medicines they needed. Similar qualitative work has shown that long‐term medication use for diabetes and hypertension is influenced by both health‐system barriers and patient perceptions of chronic disease [28]. Secondary analysis of NCD medication adherence in India also indicates that service access and socioeconomic constraints are important determinants of adherence behaviour [29]. Occupation was also associated with medication adherence. Housewives had significantly higher adjusted odds of low adherence than retired participants. Comparable findings have been reported among patients with hypertension and diabetes, where housewives demonstrated poorer medication adherence than other occupational groups [30]. This association should not be interpreted as evidence that being a housewife causes low adherence. It may reflect differences in household responsibilities, time constraints, socioeconomic circumstances, or access to healthcare, although these explanations were not directly established by the present study. Although sex itself was not significantly associated with adherence in this study, the occupational pattern suggests that domestic roles may create specific barriers to regular medicine use. The inconsistency in the association between occupation and medication adherence across studies may reflect differences in study populations, employment characteristics, and sociocultural contexts. This interpretation is consistent with evidence that patient beliefs, family responsibilities, and social context influence medication adherence among patients with chronic cardiometabolic conditions [31]. Work pressure was independently associated with low adherence. Patients with irregular or demanding work may miss doses, delay follow‐up, or be unable to attend NCD Corners during facility hours. This finding is supported by recent Bangladeshi evidence identifying busy work schedules as one of the most frequently reported reasons for missed antihypertensive medication, highlighting occupational demands as a persistent behavioural barrier to long‐term treatment adherence [25]. Practical strategies such as flexible refill arrangements, longer prescription duration for clinically stable patients, reminder systems, and decentralised refill mechanisms may reduce work‐related missed doses [31]. These approaches should be considered potential service improvements rather than interventions whose effectiveness was evaluated in this study. Together, these findings suggest that adherence interventions should combine patient‐centred behavioural support with service delivery approaches that better accommodate patients' occupational and household responsibilities. These measures may be particularly useful in rural and sub‐district settings where patients must balance healthcare visits with daily livelihood responsibilities. Distance from the NCD Corner was significantly associated with low adherence in bivariate analysis but was not significant after adjustment (AOR 1.43, 95% CI 0.80–2.54; *p* = 0.228). Although the proportion with low adherence was higher among participants living more than 5 km from an NCD Corner than among those living within 5 km (64.9% vs. 47.4%), this association did not remain statistically significant in the adjusted model. This suggests that the effect of distance may be partly mediated by transport cost, work pressure, medicine availability, and whether the facility visit results in complete prescription fulfilment. Evidence from West Bengal has similarly shown that distance from healthcare facilities can affect adherence to treatment regimens [32]. The loss of significance after adjustment suggests that the observed association between distance from the NCD Corner and medication adherence may be partly explained by other factors included in the model. However, given the cross‐sectional design, the underlying pathways cannot be established. This finding should be interpreted cautiously in light of evidence from Bangladesh and a scoping review of medication adherence among patients with hypertension and diabetes [7, 33]. Evidence from rural Bangladesh also identifies distance‐related access barriers as a contributor to poor medication adherence [14]. Education level, disease status, and disease duration were not independently associated with adherence after adjustment. Patients with both hypertension and diabetes showed slightly better adherence in descriptive analysis, but this pattern did not remain statistically significant. Systematic evidence on hypertension adherence shows that determinants vary across populations and are often shaped by interacting behavioural, clinical, and service factors [34]. Similarly, recent studies from Bangladesh and rural India found no independent association between education and medication adherence after adjustment [25, 35]. Although a nationwide Bangladeshi study reported lower adherence among hypertensive patients with coexisting diabetes [33], this association was not observed in the present study, possibly reflecting differences in study settings and the stronger influence of health‐system factors. These findings align with evidence that adherence is shaped by the combined effects of patient‐, therapy‐, and health‐system determinants [7]. Similar variation has been observed among patients with type 2 diabetes in other settings [36]. From a programmatic perspective, the findings point to three levels of intervention. First, NCD Corners require reliable medicine supply chains with buffer stock, routine stock monitoring, and timely replenishment. Second, prescription fulfilment should be monitored because partial dispensing was strongly associated with low adherence. Third, patient counselling should emphasise lifelong treatment, consequences of missed doses, and practical strategies for overcoming forgetfulness and work‐related barriers. Broader evidence on chronic disease medication adherence supports multifaceted interventions that address both individual behaviour and health‐system constraints [37]. In rural contexts, socioeconomic barriers should also be considered when designing adherence‐support interventions [5]. In addition, feasible refill arrangements that reduce unnecessary travel and accommodate patients' work and household responsibilities could be considered. The suitability and effectiveness of these approaches should be evaluated in future implementation studies.

The present study extends the evidence on medication adherence in Bangladesh by demonstrating that both patient‐ and health‐system factors were associated with adherence among patients receiving care through primary healthcare NCD Corners. The mixed‐methods design provided a comprehensive understanding of the barriers to sustained medication use by integrating quantitative findings with the experiences of patients and healthcare providers. The strong associations observed for incomplete or absent medicine dispensing highlight the importance of considering whether patients can obtain their prescribed medicines, rather than viewing adherence only as an individual behavioural responsibility. However, the cross‐sectional design does not establish causality, and the wide confidence interval for the association with receiving no medicines indicates uncertainty in the magnitude of that estimate. The findings suggest that improving medicine availability and prescription fulfilment, alongside interventions addressing behavioural barriers such as forgetfulness and work‐related constraints, may strengthen medication adherence within routine NCD services. Future longitudinal and implementation studies are needed to evaluate the effectiveness of these integrated strategies in improving long‐term adherence and clinical outcomes.

## 5 Strengths and Limitations

A key strength of this study is its mixed‐methods design, which combined quantitative assessment of medication adherence with qualitative insights from patients and healthcare providers, enabling a more comprehensive understanding of adherence barriers. The use of the validated MMAS‐8 provided a standardised assessment of medication adherence, while multivariable logistic regression strengthened the identification of independent predictors. The study also evaluated patients with hypertension, diabetes, and both conditions within government NCD Corners, enhancing its relevance to primary healthcare policy and practice in Bangladesh.

The study has several limitations. It was conducted in selected NCD Corners within one district, which may limit the generalisability of the findings to other healthcare settings in Bangladesh. The achieved sample size was smaller than planned, which may have reduced statistical precision and the ability to detect associations, particularly in small subgroups. Medication adherence was self‐reported and may therefore be affected by recall and social desirability bias, as objective adherence measures were unavailable. The cross‐sectional design precludes causal inference and assessment of changes in adherence over time. In addition, participants were recruited only from patients attending NCD Corners, introducing the possibility of selection bias. Variables selected based on bivariate significance may have excluded important confounders, resulting in residual confounding and requiring cautious interpretation of adjusted associations. Finally, some regression estimates had wide confidence intervals because of small sample sizes in certain categories, reducing the precision of those estimates. Data saturation was not assessed, and participants were not asked to verify transcript accuracy or the researchers' interpretations. In particular, the wide confidence interval for receiving no medication indicates substantial uncertainty, and this estimate should be interpreted cautiously.

## 6 Conclusion

Low medication adherence was common among patients with hypertension and/or diabetes attending NCD Corners at sub‐district hospitals in Gopalganj, Bangladesh. The findings suggest that low adherence was associated with both patient‐related factors and health‐system barriers, particularly inadequate medicine availability and incomplete prescription fulfilment. Housewives, patients missing doses because of work pressure, and those receiving incomplete or no prescribed medicines from NCD Corners were especially vulnerable to low adherence. These findings provide evidence to support strengthening the delivery of primary healthcare NCD services in Bangladesh through reliable medicine supply chains, complete prescription fulfilment, enhanced adherence counselling, and patient‐friendly refill systems to improve medication adherence and continuity of care. Future implementation and longitudinal studies are warranted to evaluate the effectiveness of these strategies in improving long‐term medication adherence and related health outcomes.

## Author Contributions

**Sabrina Mohona:** conceptualisation, investigation, writing – original draft, methodology, validation, visualisation, writing – review and editing, formal analysis, project administration, data curation, supervision. **Md. Ahashan Habib:** conceptualisation, investigation, writing – original draft, methodology, validation, visualisation, writing – review and editing, formal analysis, project administration, data curation, supervision. **Ifteker Uddin Sakib:** conceptualisation, investigation, writing – original draft, writing – review and editing, visualisation, validation, methodology, formal analysis, data curation. **Urmila Rahman:** methodology, validation, visualisation, writing – original draft, formal analysis, data curation. **Tahmina Shirin:** validation, visualisation, writing – original draft, formal analysis, data curation. **Quazi Ahmed Zaki:** validation, visualisation, data curation, formal analysis, writing – original draft. **Rashedul Hasan:** validation, visualisation, writing – original draft, data curation.

## Funding

The authors have nothing to report.

## Ethics Statement

Ethical approval was obtained from the Institutional Review Board (IRB) of the Institute of Epidemiology, Disease Control and Research (IEDCR), Bangladesh (Memo No. IEDCR/IRB/2021/04). Written or verbal informed consent, according to approved study procedures, was obtained from all participants before data collection.

## Consent

Verbal or written informed consent for publication of anonymised data was obtained from all participants.

## Conflicts of Interest

The authors declare no conflicts of interest.

## Transparency Statement

The lead author and corresponding author affirm that this manuscript is an honest, accurate, and transparent account of the study being reported; that no important aspects of the study have been omitted; and that any discrepancies from the study as planned have been explained.

## Data Availability

The data that support the findings of this study are available from the corresponding author upon reasonable request.
